# Delivery of four‐dimensional radiotherapy with TrackBeam for moving target using a dual‐layer MLC: dynamic phantoms study

**DOI:** 10.1120/jacmp.v10i2.2926

**Published:** 2009-04-23

**Authors:** Yaxi Liu, Chengyu Shi, Bryan Lin, Chul Soo Ha, Niko Papanikolaou

**Affiliations:** ^1^ University of Texas Health Science Center Radiation Oncology Department San Antonio TX USA

**Keywords:** beam tracking, dual‐layer MLC, fiducial marker, respiratory motion

## Abstract

Respiratory motion has been considered a clinical challenge for lung tumor treatments due to target motion. In this study, we aimed to perform an experimental evaluation based on dynamic phantoms using MLC‐based beam tracking. TrackBeam, a prototype real‐time beam tracking system, has been assembled and evaluated in our clinic. TrackBeam includes an orthogonal dual‐layer micro multileaf collimator (DmMLC), an on‐board mega‐voltage (MV) portal imaging device, and an image processing workstation. With a fiducial marker implanted in a moving target, the onboard imaging device can capture the motion. The TrackBeam workstation processes the online MV fluence and detects and predicts tumor motion. The DmMLC system then dynamically repositions each leaf to form new beam apertures based on the movement of the fiducial marker. In this study, a dynamic phantom was used for the measurements. Three delivery patterns were evaluated for dosimetric verification based on radiographic films: no‐motion lung‐tumor (NMLT), three‐dimensional conformal radiotherapy (3DCRT), and four‐dimensional tracking radiotherapy (4DTRT). The displacement between the DmMLC dynamic beam isocenter and the fiducial marker was in the range of 0.5 mm to 1.5 mm. With radiographic film analysis, the planar dose histogram difference between 3DCRT and NLMT was 48.6% and 38.0% with dose difference tolerances of 10% and 20%, respectively. The planar dose histogram difference between 4DTRT and NLMT was 15.2% and 4.0%, respectively. Based on dose volume histogram analysis, 4DTRT reduces the mean dose for the surrounding tissue from 35.4 Gy to 19.5 Gy, reduces the relative volume of the total lung from 28% to 18% at V20, and reduces the amount of dose from 35.2 Gy to 15.0 Gy at D20. The experimental results show that MLC‐based real‐time beam tracking delivery provides a potential solution to respiratory motion control. Beam tracking delivers a highly conformal dose to a moving target, while sparing surrounding normal tissue.

PACS number: 87.55.de, 87.55.ne, 87.56.nk

## I. INTRODUCTION

Tumor motion, especially that caused by respiration, introduces technical challenges for radiation treatment delivery. Management of respiratory motion has been outlined in the report of the American Association of Physicists in Medicine (AAPM) Task Group 76.^(1)^ Respiratory motion affects tumor sites in the thorax and abdomen, resulting in tumor movement on the order of several centimeters. This compromises the benefits of highly conformed dose delivery with three‐dimensional conventional radiotherapy (3DCRT) or intensity modulated radiotherapy (IMRT) techniques.^(^ ^1^ ^–^ ^3^ ^)^ Differences between planned and delivered radiation treatments may occur due to the respiration‐induced tumor motion, leading to insufficient dose to the tumor and excess dose to the surrounding normal structures.^(^ ^4^ ^–^ ^5^ ^)^ Existing methods that attempt to account for tumor motion can be grouped into two categories: discrete delivery methods (such as gating), and continuous delivery methods, known as beam tracking.^(^ ^6^ ^–^ ^10^ ^)^

For gating technology, the moving target is expected to be at a certain position during a specific interval of the respiratory cycle. The position and width of the gate within a respiratory cycle is determined by monitoring the patient's respiratory motion.^(^ ^1^ ^,^ ^11^ ^–^ ^14^ ^)^ Typically, an external marker may be placed halfway between the xiphoid and the umbilicus, or an internal fiducial marker may be implanted into the patient's body. Respiratory‐gated radiation delivery has been studied by several institutions.^(^ ^8^ ^,^ ^15^ ^–^ ^16^ ^)^ In this method, a threshold is applied to define a gating window based on the respiratory signal, and the beam is turned on for the period of time when the target is within the desired window. Because beam delivery is not continuous, gated procedures are longer than non‐gated procedures. Generally, a gating duty cycle of 30% to 50% leads to an increase in delivery time by a factor of 2 to 3.^(17)^ ^,^ ^(18)^ For one feasibility study reported in the literature, the respiratory gating increased the delivery time by a factor of 4 to 15.^(^ ^1^ ^,^ ^18^ ^–^ ^19^ ^)^

Another solution to accommodate tumor motion is to use the beam to track tumor motion.^(^ ^1^ ^,^ ^20^ ^–^ ^21^ ^)^ Target tracking techniques use dynamic beam repositioning to follow the tumor position in real time. Theoretically, tracking provides an ideal solution to tumor motion by eliminating the need for a tumor motion margin and utilizing the full duty cycle of dose delivery.^(1)^ The tumor tracking methods have their own clinical challenges including determining the tumor position, repositioning the beam, and synchronizing the beam delivery with tumor motion.^(20)^ ^,^ ^(21)^

The CyberKnife (Accuray, Sunnyvale, CA) tracking system introduced by Schweikard et al.^(22)^ is an example of a system that has beam tracking capabilities. The motion of the robotically‐controlled linac is synchronized to the patient's external respiratory motion, allowing it to follow tumor motion in real time. Keall et al.^(20)^ extracted motion envelopes from 4D CT scans and applied them to modify dynamic multileaf collimator (MLC) movements, thereby showing the feasibility of MLC‐based adaptive tracking. A couch‐based approach by D'Souza et al.^(9)^ showed similar feasibility, allowing a clearer distinction of beam shaping and motion compensating movements. Neuci et al.^(23)^ developed a technique to synchronize the moving radiation beam aperture with the tumor motion induced by respiration.

In this study, an MLC‐based beam tracking system, TrackBeam (Initia Ltd, Israel) has been assembled and evaluated in our clinic. TrackBeam utilizes a dual‐layer micro MLC (DmMLC) in combination with an online megavoltage (MV) portal imaging device (PID) and a gold fiducial marker implanted inside the tumor.^(24)^ ^,^ ^(25)^ The MV PID determines the target position in real time by processing the fiducial marker projection (FMP). The dual‐layer MLC, offering orthogonal upper and lower banks of leaves, was used to reposition the beam aperture in two dimensions simultaneously. As a result, the dual‐layer MLC provided less angular dependence compared with a standard single‐layer MLC.^(24)^ ^,^ ^(25)^ An integrated image processing tool (IPT) with an embedded adaptive functionality compensated for the latency of the dynamic beam isocenter and fiducial marker projection. In this study, we have investigated real‐time beam tracking delivery based on dynamic phantoms. We present our results comparing this device with conventional delivery and tracking delivery patterns.

## II. MATERIALS AND METHODS

### A. An MLC‐based real‐time Beam Tracking System

Beam tracking in radiation therapy is a delivery mode whereby a dynamic MLC tracks tumor motion in real time. Single or multiple implanted fiducial markers can be used to show tumor motion. Real‐time images can be obtained, processed, and transferred to a dynamic multileaf collimator (MLC) control unit. The dynamic MLC reshapes the beam aperture according to the fiducial marker motion. Challenges of beam tracking include detecting the tumor position in real time and synchronizing the MLC beam aperture with the motion of the target.

**Figure 1:**
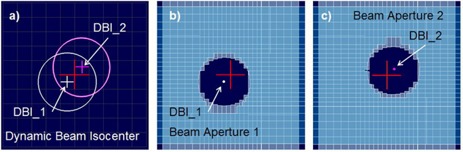
Schematic layout of MLC‐based beam tracking delivery for motion tumor: (a) dynamic beam isocenter; (b‐c) dynamic beam apertures corresponding to dynamic beam isocenter

Figure 1(a) illustrates the schematic layout of dynamic MLC‐based beam tracking system. A dynamic beam isocenter (DBI) was used as the reference point for the dynamic beam to reposition each leaf and form a new beam aperture. For instance, a round beam aperture was formed to cover a spherical shape target, in which the DBI was set to the center of the target. As the DBI moves, each MLC leaf is repositioned to generate new beam apertures. Figures 1(b) and 1(c) show two beam apertures created for a moving DBI. The motion of the DBI was determined by the motion of a pre‐implanted fiducial marker.

**Figure 2:**
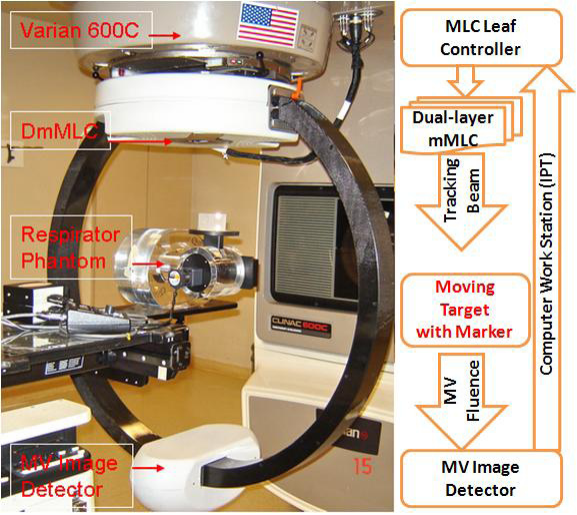
4D tracking radiotherapy experimental settings. The right side displays the process path of 4DTRT.

TrackBeam integrates an image processing tools (IPT) workstation, a dual‐layer micro MLC, and a portal imaging device. Figure 2 shows the beam tracking setup for delivery to a moving target within a dynamic phantom. A DmMLC and an MV PID were attached to a Varian 600C linear accelerator (Varian Medical Systems, Inc., Palo Alto, CA, USA). A Quasar (Modus Medical Devices, Inc. London, ON Canada) respiratory phantom was used to simulate target motion due to breathing.

The IPT workstation processed the online MV fluence images, detected the fiducial marker projection, and controlled the MLC leaf motion. The fiducial marker motion was captured by a CV‐M300 industrial monochrome charge‐coupled device (CCD) camera (JAI Corporation, Kanagawa, Japan). A frame grabber was used to capture the fiducial marker as the reference image, which was then processed by Sapera (DALSA Corporation, Waterloo, Ontario, Canada). Each leaf of the DmMLC was controlled by a separate motor traveling at maximum speed of 20 mm/sec. The orthogonal layers of the DmMLC reduce MLC field dependence of the leaf stepping angle and provide fast beam response.^(24)^ ^,^ ^(25)^ Both factors are important in MLC‐based beam tracking.

The latency between the DBI and fiducial markers is a critical factor in beam tracking delivery. A Kalman filter‐based adaptive function introduced by Kalman and Bucy in the 1960s reduced the positioning lag.^(26)^ ^,^ ^(27)^ The Kalman Filter (KF) is based on a linear dynamic system in which continuous models are transferred into discrete counterparts in the time domain. The KF is a predictor‐corrector type of estimator that implements a set of mathematical models and minimizes the estimated error covariance when certain conditions are met. The Kalman filter is also a recursive estimator, which means that the estimated state from the previous time step and the current measurement are needed to compute the estimate for the current state.

### B. Dynamic respiratory Phantoms and Fiducial Markers

**Figure 3:**
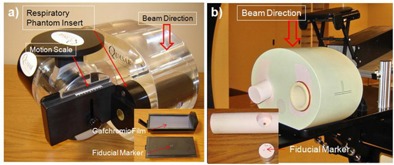
Respiratory motion phantom and fiducial markers: a) a Quasar phantom and its insert with Gafchromic EBT film and fiducial marker; b) a CIRSTM thorax tissue equivalent dynamic phantom and its insert with fiducial marker.

The Quasar respiratory motion phantom is a thorax phantom capable of moving in the superior‐inferior direction with variable speed and amplitude. Major components of this respiratory phantom system, shown in Fig. 3(a), include a thorax phantom, a precise motion actuator, and a controller with preset motion profiles. Target motion in the respiratory phantom is achieved by the application of a sinusoidal pattern of motion.

Another dynamic phantom used was the CIRSTM dynamic tissue equivalent thorax phantom, shown in Fig. 3(b). A CT of the CIRSTM phantom was used to develop 3DCRT and 4DTRT plans, and the resulting DVHs were compared.

Acculoc gold fiducial markers (CIVCO Medical Solutions, Kalona, Iowa) were used to allow localization of the target in MV images. These fiducials are 3 mm×1.0 mm gold cylinders that are specially knurled to inhibit migration. One fiducial was implanted in each phantom.

### C. Beam Tracking Delivery Experimental Measurements

#### C.1 Latency Between Dynamic Beam Isocenter and Fiducial Marker

The latency between the DBI and fiducial marker projection (FMP) was the first parameter evaluated in this study. At the beginning of a treatment, the DmMLC leaves are not perfectly synchronized with the motion of the marker, resulting in a time lag between movement of the marker and the formation of a new MLC pattern. The IPT workstation provides an adaptive function to calculate and forecast the latency between the DBI and FMP.

In beam tracking delivery, it is important to evaluate the displacement between the dynamic beam isocenter and the fiducial marker. There exists a latent period between the movement of the target and that of the beam isocenter as it tracks the target. To quantify this latency, the displacement between the DBI and the FMP at various phases were measured. The latency was then calculated using the displacement of the DBI and FMP divided by the velocity at that given phase. The relationship of the fiducial marker movement as function of time was expressed as Eq. (1).

**Figure 4:**
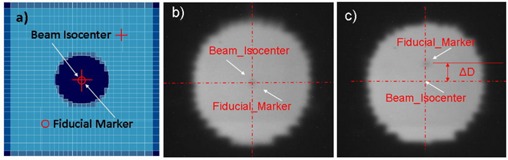
Beam's eye view (BEV) of dynamic beam and MV fluence image in beam tracking: (a) BEV of dynamic beam isocenter overlapping with fiducial marker; (b) MV fluence image with synchronized dynamic beam isocenter and fiducial marker projection; (c) MV fluence image with unsynchronized dynamic beam isocenter and fiducial marker projection.

(1) FMP(t)=Asin(ωt+θ)

where A is the fiducial marker projection amplitude, co is the angular frequency of the sinusoidal motion, t is time and 9 is the initial phase. Figure 4(a) shows the beam's eye view (BEV) of the DmMLC leaf position and the dynamic beam isocenter (DBI). For the stationary lung tumor, we overlapped the beam isocenter with the fiducial marker at the center of the circular shape. Figures 4(b) and (c) were taken from the MV fluence video recorded by the PID using Sapera, with a recording rate of 12.5 frames per second. Figure 4(b) represents the ideal beam tracking case, in which the beam aperture formed by the DmMLC leaves is synchronized with the marker position. Figure 4(c) shows a scenario where the beam isocenter did not synchronize with the fiducial marker motion.

The displacement between the DBI and FMP is shown by Eq. (2):

(2) ΔD(t)=DBI(t)−FMP(t)

where the DBI(t) is the location of the dynamic beam isocenter relative to the original isocenter as a function of time. Therefore, the time latency can be obtained by dividing ΔD(t) by the marker's velocity V(t):

(3) ΔT=ΔD(t)/V(t)

where velocity, V(t),is the derivative of FMP(t):

(4) V(t)=Aωcos⁡(ωt+θ)

When the marker velocity is close to zero (i.e. at the end of inhalation and the end of exhalation), Eq. (3) is no longer satisfied. In this situation, the latency was estimated by averaging the latency of two adjacent phases.

### C.2 radiographic Films in Beam Tracking Delivery

In order to evaluate the dosimetric effect of beam tracking, we performed radiographic film comparisons using Gafchromic (International Specialty Products, NJ, USA) film inside the Quasar phantom for three delivery patterns (NMLT, 3DCRT, and 4DTRT). The NMLT case was an ideal scenario in which the lung tumor was assumed to be stationary and was used as the reference pattern to compare with 3DCRT and 4DTRT. In 3DCRT, an additional margin of 1.0 cm to 1.5 cm was added to the clinical tumor volume to account for tumor motion. For the evaluated 4DTRT, the same beam aperture was used with NMLT pattern, but with no additional margin. Both dose profiles and planar doses were analyzed for these three evaluated patterns.

## III. RESULTS

The respiratory phantom was set to travel from the end‐inhale phase to the end‐exhale phase at a distance of 20 mm, with respiratory rate as 5 s/cycle.

### A. Latency Measurement Between MLC Virtual Isocenter and Fiducial Marker

The real‐time tracking system was found to have a latency time of 100 ms during the first one to two cycles. The synchronization of DBI to FMP was achieved after approximately three to four cycles.

**Figure 5:**
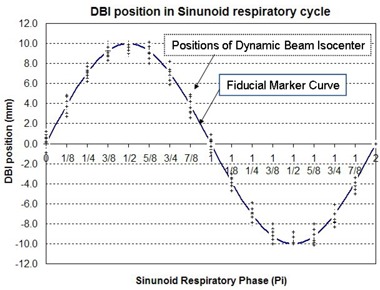
Dynamic beam isocenter and fiducial marker motion curve of a sinusoidal motion cycle at 16 phases over 12 respiratory cycles.

To measure the time difference between the dynamic beam aperture formation and position of the moving marker, 16 phases were selected for each sinusoidal cycle. In the DBI measurements, the fiducial marker motion amplitude was set to 20 mm with a respiratory period of 5.0 s/cycle. Figure 5 shows the position of DBI at each phase over 12 respiratory cycles.

The DBI measurements and the fiducial marker calculation are listed in Table I. The target has very little instantaneous velocity at the end of inhalation and at the end of exhalation. For these cases, the latency was estimated by averaging two adjacent phases.

**Table 1:** Average latency of the DBI and FMP for a sinusoid cycle.

*Phase #*	*Radian (n)*	*FMP(t) (mm)*	*DBI (t) (mm) Averaged 12 cycles* ^a^	*SD (mm) Averaged 12 cycles*	*ΔD (t) (mm)*	*V (t) (mm/sec)*	*Latency (ms)* ^b^
P0	0	0.0	0.3	0.7	0.3	12.6	21
P1	1/8	3.9	3.8	0.6	0.0	11.6	3
P2	1/4	7.1	7.1	0.6	0.1	8.9	6
P3	3/8	9.3	9.3	0.6	0.0	4.8	5
P4	1/2	10.0	9.4	0.4	0.6	0.0	35 (est.)
P5	5/8	9.2	8.9	0.6	0.3	4.8	61
P6	3/4	6.9	6.8	0.6	0.1	8.9	7
P7	7/8	3.6	3.8	0.7	0.2	11.6	15
P8	1	‐0.3	0.0	0.5	0.3	12.6	22
P9	1 1/8	‐4.2	‐3.5	0.6	0.7	11.6	59
P10	1 1/4	‐7.3	‐7.1	0.6	0.2	8.9	25
P11	1 3/8	‐9.4	‐9.0	0.5	0.4	4.8	74
P12	1 1/2	‐10.0	‐9.5	0.4	0.5	0.0	40 (est.)
P13	1 5/8	‐9.0	‐9.1	0.6	0.0	4.8	7
P14	1 3/4	‐6.7	‐7.3	0.6	0.6	8.9	65
P15	1 7/8	‐3.3	‐3.9	0.6	0.7	11.6	57

a Averaged measurements of DBI for 12 respiratory cycles after the DBI was synchronized with the FMP.

b Marker's momentary velocity at the end of inhalation and the end of exhalation is very low, so the latency was estimated using the average latency of two adjacent phases.

### B. Dosimetric Analysis of radiographic Films

**Figure 6:**
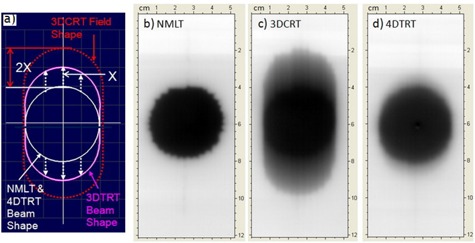
Films of the three evaluated patterns: (a) layout of the 3DCRT pattern; (b) NMLT; (c) 3DCRT; (d) 4DTRT.

Figure 6(a) shows a schematic layout of a 3DCRT plan for a mobile tumor. To account for the target motion, an additional margin is needed along the direction of target motion to ensure adequate coverage of the target. The superior–inferior (S–I) direction is the axis of greatest motion for most patients.^(28)^ An elliptical target was obtained by giving an additional X margin to both sides of the target along the target motion direction. The outer field shape (dashed line) was the dose delivered to the moving target and its surrounding tissue. When the tumor moved by a distance of X, the field shape was extended by a distance of up to 2X.

The 4DTRT test cased utilized real‐time tracking beam to account for tumor motion. The DmMLC was set to generate a 4.0 cm circular aperture with the beam perpendicular to the plane of motion. The tumor motion was simulated by a 5.0 s/cycle sinusoidal pattern with an amplitude of ±10 mm in the superior‐inferior (SI) direction.

Figures 6(b) to (d) show the results of the analysis of Gafchromic EBT films for the NMLT, 3DCRT and 4DTRT patterns. With 3DCRT, the field was blurred due to tumor motion and resulted in a distorted shape along the direction of motion. The field delivered with 4D tumor tracking was rounded and had much less blur compared to the 3DCRT pattern. The 4DTRT provided a dose distribution that closely matched the pattern that could be expected in the absence of tumor motion.

**Figure 7:**
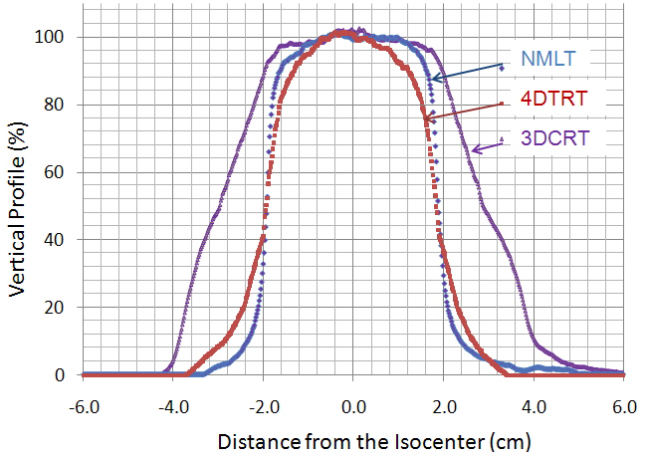
Radiographic films of the three evaluated patterns: (a) NMLT; (b) 3DCRT; (c) 4DTRT.

Figure 7 shows the vertical profiles of the 3DCRT, 4DTRT and NMLT patterns. The 3DCRT plan overdosed surrounding tissues because an additional margin had to be added to accommodate the target motion. The 4DTRT overdosed a small percentage of tissue outside the field edge and underdosed a small percentage of the tumor due to beam latency. The profile analysis of the three patterns demonstrated that 4DTRT better approximates the NMLT case than does the 3DCRT plan. To get quantitative results, additional analyses, including planar dose differences, were applied to compare the dose characteristics of the three patterns.

Planar dose subtraction was used to compare the difference between two dose distributions. The difference plot is based on pixel by pixel subtraction of two co‐registered images. This type of plot is not only capable of showing the amplitude of the dose difference between two images, but also the location of the difference.

**Figure 8:**
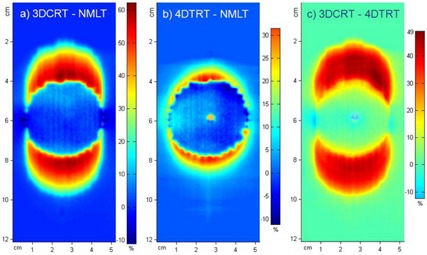
Planar dose difference percentages of co‐registered calibrated films: (a) 3DCRT – NMLT; (b) 4DTRT – NMLT; (c) 3DCRT – 4DTRT.

Figures 8(a) to (c) show the planar dosimetric comparison of the co‐registered images from the NMLT, 3DCRT and 4DTRT patterns. All the three patterns were based on the same planning tumor volume, which was a 4 cm diameter sphere. For the NMLT and 4DTRT cases, the same circular beam apertures (4 cm in diameter) were used. For the 3DCRT, an elliptical aperture was created using 1 cm additional margin extended from the round beam shape along the fiducial marker motion direction.

Figure 8(a) shows that 3DCRT induced an unavoidable over‐delivery of dose to the surrounding tissue. Compared to the 3DCRT, 29.9% of the NMLT total pixel count exceeded the desired dose difference of ±5%, with a maximum dose difference of 69.0%. Figure 8(b) shows the dosimetric comparison of the 4DTRT and NMLT pattern. The 4DTRT pattern delivers conformal dose to the tumor with less than 5% difference in dose when compared to the NMLT case. In addition, less surrounding tissue received excess dose. Figure 8(c) directly compares the planar dose between the 3DCRT and the 4DTRT patterns.

**Figure 9:**
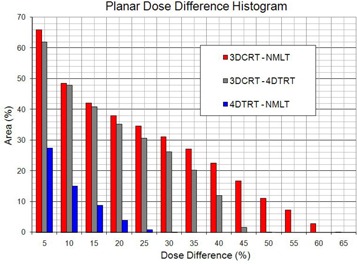
Planar dose percent difference histograms of co‐registered calibrated films: (a) 3DCRT – NMLT; (b) 4DTRT – NMLT; (c) 3DCRT – 4DTRT.

To quantify the dose difference of these three evaluation patterns, we selected a region of interest (ROI) based on the isodose line of the 3DCRT flm. A rectangle with dimensions of 4.4 cm×7.75 cm was selected for all the three radiographic films. The planar dose difference histogram of the 3DCRT, 4DTRT and NMLT patterns are shown in Table II. The dosimetric analysis of radiographic films show 48.6%, 38.0%, 31.1% 22.5% and 11.1% of pixels exceeding the dose difference tolerance of 10%, 20%, 30%, 40% and 50%, respectively, when comparing the 3DCRT pattern to the NMLT pattern. Comparing the film of 4DTRT to the NNLT, 15.2% and 4.0% of pixels exceeding the dose difference tolerance were measured with dose difference tolerance at 10% and 20%, respectively. When comparing 4DTRT and NMLT patterns, 27.5% of the pixels of the co‐registered images exceeded the selected dose difference tolerance of 5.0%. Figure 9 shows the dose histogram difference of co‐registered calibrated films of the NMLT, 3DCRT, and 4DTRT patterns.

**Table 2:** Planar dose difference histogram of the patterns of 3DCRT, 4DTRT and NMLT.

			*Percentage of pixels exceeding the dose difference tolerance*									
*Tolerance (%)*	*5*	*10*	*15*	*20*	*25*	*30*	*35*	*40*	*45*	*50*	*55*	*60*
3DCRT vs. NMLT	65.8	48.6	42.0	38.0	34.6	31.1	27.2	22.5	16.7	11.1	7.3	2.9
3DCRT vs. 4DTRT	61.9	47.8	40.8	35.3	30.7	26.2	20.3	12.0	1.7	‐	‐	‐
4DTRT vs. NMLT	27.5	15.2	8.9	4.0	0.9	‐	‐	‐	‐	‐	‐	‐

## IV. DISCUSSION

### A. DVH Comparison of a 3DCrT and a 4DTrT Planning to the CIrSTM Dynamic Phantom

**Figure 10:**
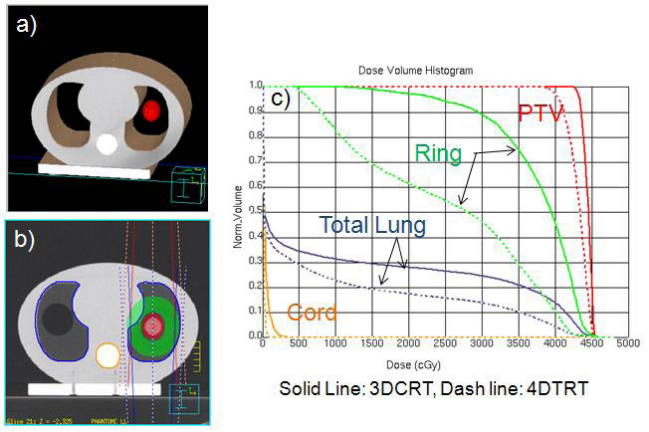
Tissue equivalent dynamic phantom: (a) 3D view of lung phantom with tumor in left lung; (b) AP/PA beam setting with total lung, cord, ring, and target contours; (c) DVH comparison for the 3DCRT and the 4DTRT planning.

The CIRSTM dynamic phantom was scanned in the LightSpeed CT scanner (GE Healthcare, USA) with a slice thickness of 2.5 mm (120 kVp and 80 – 440 mA). A treatment plan was developed using anterior/posterior (AP) and posterior/anterior (PA) beam arrangements. A ring‐shaped structure was created at a radius of 2.5 cm outside the PTV to allow assessment of the dose delivered to surrounding healthy tissue. An additional margin of 1.5 cm was added, accounting for the tumor motion with the 3DCRT compared with 4DTRT, as shown in Fig. 10.

The DVH comparisons indicate that 4DTRT reduces the dose to the ring structure from 97.5% to 61.5% of the volume at V20, and reduces the dose to 80% of the volume (D80) from 33.5 Gy to 11.5 Gy. 4DTRT also reduces the relative volume of the total lung from 28% to 18% at V20, and reduces the dose from 35.2 Gy to 15.0 Gy at D20. The lung V5, V10, and V20 were 35.0%, 31.0% and 28.0%, respectively, for the 3DCRT for a prescribed dose of 45 Gy. For the 4DTRT delivery, the lung V5, V10, and V20 were 30.0%, 22.0% and 18.0%, respectively. The mean dose to the ring was 35.4 Gy for the 3DCRT, and 19.5 cGy for the 4DTRT.

### B. rigid/Deformable Target

We have implemented beam tracking delivery to compensate for rigid tumor motion in a phantom‐based experiment. To implement this technique in a clinical setting, we must assume a rigid transformation of the PTV with no deformation of the PTV. One case in which this may be appropriate has been reported by Deurloo^(29)^ who found that the prostate exhibits shape deformations that are small relative to its motion. A first order correction is therefore feasible for setup errors and organ motion only.

Traditionally, tumor movement has been compensated for by applying greater margins so that the tumor is always covered by the beam. Studies in the lung by Engelsman and Beckham^(30)^ ^,^ ^(31)^ have both found dose errors of 5%. However, the change in equivalent uniform dose was 0.5%. Therefore, it could be argued that for realistic organ displacements and multiple field treatments, the dose error is small with the appropriate algorithm. This renders the delivered dose invariant to tissue displacement, as long as full coverage of the tumor is achieved.^(32)^

Testing of periodic asymmetric motion tracking, as exhibited by many organs affected by breathing (such as lung and liver), and further testing of realistic motion patterns are needed to understand and account for motion drift over prolonged periods of treatment. Evaluation of the effects of rapid changes of motion, such as that caused by coughing, will be conducted in the future.

## V. CONCLUSIONS

MLC‐based beam tracking delivery provides an opportunity to achieve conformal coverage of moving tumors and reduce the dose to the surrounding normal tissue by reducing target margins. The dosimetric analysis between the 3DCRT and NMLT experiments indicate that a total of 48.6%, 38.0%, 31.1%, 22.5%, and 11.1% of the pixels in the region of interest (ROI) of the dose image exceed the dose difference tolerances of 10%, 20%, 30%, 40% and 50%, respectively. However, when comparing the 4DTRT to the NMLT, only 15.2% and 4.0% of the pixels exceeded the dose difference tolerances of 10% and 20%, respectively. The DVH comparisons indicate that 4DTRT reduces the V20 of the surrounding tissue from 97.5% to 61.5%, and reduces the D80 from 33.5 Gy to 11.5 Gy. 4DTRT also reduces the amount of dose to the total lung from 28% to 18% of the total lung volume at V20, and reduces the D20 from 35.2 Gy to 15.0 Gy.

The beam tracking method examined in this study compensated for the tumor motion in two dimensions, providing improvement in both dose distribution and coverage while reducing the dose to surrounding tissue. Deformable targets and IMRT implementation of 4DTRT are forthcoming as an extension of our current study.

## ACKNOWLEDGEMENTS

This work was partially supported by Grants from Initia Ltd. and a National Institutes of Health/National Library of Medicine grant (1R01LM009362‐01). The authors would like to acknowledge Dr. Shifeng Chen (Duke University) and Courtney Buckey (University of Texas Health Science Center) for their help on the manuscript.

## Supporting information

Supplementary Material

Supplementary Material

## References

[1] Keall PJ , Mageras GS , Balter JM , et al. The management of respiratory motion in radiation oncology: report of AAPM Task Group 76. Med Phys. 2006;33(10):3874–3900. 17089851 10.1118/1.2349696

[2] Shirato H , Oita M , Fujita K , Shimizu S , et al. Three‐dimensional conformal setup (3D‐CSU) of patients using the coordinate system provided by three internal fiducial markers and two orthogonal diagnostic X‐ray systems in the treatment room. Int J Radiat Oncol Biol Phys. 2004;60(2):607–12. 15380598 10.1016/j.ijrobp.2004.05.042

[3] Brandner ED , Wu A , Chen H , et al. Abdominal organ motion measured using 4D CT. Int J Radiat Oncol Biol Phys. 2006;65(2):554–60. 16690437 10.1016/j.ijrobp.2005.12.042

[4] Chi PC , Mawlawi O , Nehmeh SA , et al. Design of respiration averaged CT for attenuation correction of the PET data from PET/CT. Med Phys. 2007;34(6):2039–47. 17654907 10.1118/1.2733810

[5] Guckenberger M , Wilbert J , Krieger T , et al. Four‐dimensional treatment planning for stereotactic body radiotherapy. Int J Radiat Oncol Biol Phys. 2007;69(1):276–85. 17707282 10.1016/j.ijrobp.2007.04.074

[6] Kubo HD , Wang L . Introduction of audio gating to further reduce organ motion in breathing synchronized radiotherapy. Med Phys. 2002;29(3):345–50. 11929017 10.1118/1.1448826

[7] Jiang SB , Pope C , Al Jarrah KM , Kung JH , Bortfield T , Chen GT . An experimental investigation on intra‐fractional organ motion effects in lung IMRT treatments. Phys Med Biol. 2003;48(12):1773–84. 12870582 10.1088/0031-9155/48/12/307

[8] George R , Ramakrishnan V , Siebers JV , Chung TD , Keall PJ . Investigation of patient, tumour and treatment variables affecting residual motion for respiratory‐gated radiotherapy. Phys Med Biol. 2006;51(20):5305–19. 17019040 10.1088/0031-9155/51/20/015

[9] D'Souza WD , Naqvi SA , Yu CX . Real‐time intra‐fraction‐motion tracking using the treatment couch: a feasibility study. Phys Med Biol. 2005;50(17):4021–33. 16177527 10.1088/0031-9155/50/17/007

[10] Qiu P , D'Souza WD , McAvoy TJ , Ray Liu KJ . Inferential modeling and predictive feedback control in real‐time motion compensation using the treatment couch during radiotherapy. Phys Med Biol. 2007;52(19):5831–54. 17881803 10.1088/0031-9155/52/19/007

[11] Li XA , Stepaniak C , Gore E . Technical and dosimetric aspects of respiratory gating using a pressure‐sensor motion monitoring system. Med Phys. 2006;33(1):145–54. 16485421 10.1118/1.2147743

[12] Kubo HD , Wang L . Introduction of audio gating to further reduce organ motion in breathing synchronized radiotherapy. Med Phys. 2002;29(3):345–50. 11929017 10.1118/1.1448826

[13] Timinger H , Krueger S , Borgert J , Grewer R . Motion compensation for interventional navigation on 3D static roadmaps based on an affine model and gating. Phys Med Biol. 2004;49(5):719–32. 15070198 10.1088/0031-9155/49/5/005

[14] Wang Z , Willett CG , Yin FF . Reduction of organ motion by combined cardiac gating and respiratory gating. Int J Radiat Oncol Biol Phys. 2007;68(1):259–66. 17321071 10.1016/j.ijrobp.2006.11.057

[15] Dietrich L , Tucking T , Nill S , Oelfke U . Compensation for respiratory motion by gated radiotherapy: an experimental study. Phys Med Biol. 2005;50(10):2405–14. 15876675 10.1088/0031-9155/50/10/015

[16] Keall PJ , Chang M , Benedict S , Thames H , Vedam SS , Lin PS . Investigating the temporal effects of respiratory‐gated and intensity‐modulated radiotherapy treatment delivery on in vitro survival: an experimental and theoretical study. Int J Radiat Oncol Biol Phys. 2008;71(5):1547–52. 18495369 10.1016/j.ijrobp.2008.03.047

[17] Vedam SS , Keall PJ , Kini VR , Mohan R . Determining parameters for respiration‐gated radiotherapy. Med Phys. 2001;28(10):2139–46. 11695776 10.1118/1.1406524

[18] Kubo HD , Wang L . Compatibility of Varian 2100C gated operations with enhanced dynamic wedge and IMRT dose delivery. Med Phys. 2000;27(8):1732–38. 10984218 10.1118/1.1287110

[19] Mageras GS , Yorke E . Deep inspiration breath hold and respiratory gating strategies for reducing organ motion in radiation treatment. Semin Radiat Oncol. 2004;14(1):65–75. 14752734 10.1053/j.semradonc.2003.10.009

[20] Keall PJ , Joshi S , Vedam SS , Siebers JV , Kini VR , Mohan R . Four‐dimensional radiotherapy planning for DMLC‐based respiratory motion tracking. Med Phys. 2005;32(4):942–51. 15895577 10.1118/1.1879152

[21] Ruan D , Fessler JA , Balter JM . Mean position tracking of respiratory motion. Med Phys. 2008;35(2):782–92. 18383701 10.1118/1.2825616

[22] Schweikard A , Shiomi H , Adler J . Respiration tracking in radiosurgery. Med Phys. 2004;31(10):2738–41. 15543778 10.1118/1.1774132

[23] Neicu T , Shirato H , Seppenwoolde Y , Jiang SB . Synchronized moving aperture radiation therapy (SMART): average tumour trajectory for lung patients. Phys Med Biol. 2003;48(5):587–98. 12696797 10.1088/0031-9155/48/5/303

[24] Bucciolini M , Russo S , Banci Buonamici F , Pini S , Silli P . Dosimetric characterization of a bi‐directional micro‐multileaf collimator for stereotactic applications. Med Phys. 2002;29(7):1456–63. 12148726 10.1118/1.1487423

[25] Liu Y , Shi C , Tyran P , Papanikolaou N . Dosimetric characteristics of dual‐layer multileaf collimation for small‐field and intensity‐modulated radiation therapy applications. J Appl Clin Med Phys. 2008;9(2):15–29. 10.1120/jacmp.v9i2.2709 PMC5721708 18714277

[26] Kalman RE . A new approach to linear filtering and prediction problems. Transactions of the ASME ‐ Journal of Basic Engineering. 1960;82:35–45.

[27] Kalman RE , Bucy RS . New results in linear filtering and prediction theory. Transactions of the ASME ‐ Journal of Basic Engineering. 1961;83:95–107.

[28] Alasti H , Cho YB , Vandermeer AD , et al. A novel four‐dimensional radiotherapy method for lung cancer: imaging, treatment planning and delivery. Phys. Med. Biol. 2006;(51):3251–67. 10.1088/0031-9155/51/12/017 16757875

[29] Deurloo KE , Steenbakkers RJ , Zijp LJ , et al. Quantification of shape variation of prostate and seminal vesicles during external beam radiotherapy. Int J Radiat Oncol Biol Phys. 2005;61(1):228–38. 15629616 10.1016/j.ijrobp.2004.09.023

[30] Engelsman M , Damen EM , De Jaeger K , van Ingen KM , Mijnheer BJ . The effect of breathing and set‐up errors on the cumulative dose to a lung tumor. Radiother Oncol. 2001;60;(1):95–105. 11410310 10.1016/s0167-8140(01)00349-8

[31] Beckham WA , Keall PJ , Siebers JV . A fluence‐convolution method to calculate radiation therapy dose distributions that incorporate random set‐up error. Phys Med Biol. 2002;47(19):3465–73. 12408475 10.1088/0031-9155/47/19/302

[32] Bortfield T , Jiang SB , Rietzel E . Effects of motion on the total dose distribution. Semin Radiat Oncol. 2004;14(1):41–51. 14752732 10.1053/j.semradonc.2003.10.011

